# Sublingual Adjuvant Delivery by a Live Attenuated Vibrio cholerae-Based Antigen Presentation Platform

**DOI:** 10.1128/mSphere.00245-18

**Published:** 2018-06-06

**Authors:** Julie Liao, Jacob A. Gibson, Bradley S. Pickering, Paula I. Watnick

**Affiliations:** a Division of Infectious Diseases, Boston Children’s Hospital, Harvard Medical School, Boston, Massachusetts, USA; b National Centre for Foreign Animal Diseases, Canadian Food Inspection Agency, Government of Canada, Canadian Science Centre for Human and Animal Health, Winnipeg, Manitoba, Canada; c Department of Microbiology and Immunobiology, Harvard Medical School, Boston, Massachusetts, USA; Food and Drug Administration

**Keywords:** Vibrio cholerae, cholera toxin adjuvants, live vector vaccines, mmCT, vaccine, vaccine platform

## Abstract

A sublingually delivered heterologous antigen presentation platform that does not depend on antigen or adjuvant purification would be of great benefit in protection against diarrheal disease. In proof-of-concept studies, we previously showed that when a fusion protein comprised of the Vibrio cholerae biofilm matrix protein RbmA and the B subunit of cholera toxin (R-CTB) is expressed from a plasmid within V. cholerae, R-CTB is sequestered in the biofilm matrix, leading to decoration of the cell surface. Sublingual delivery of live attenuated R-CTB-decorated cells results in a mucosal immune response to CTB. To improve the immune response to diarrheal antigens presented by this platform, we have engineered our live attenuated vaccine to express the mucosal adjuvant mmCT (i.e., multiply mutated CT). Here we report that delivery of this adjuvant via sublingual administration of our vaccine enhances the mucosal immune response to V. cholerae LPS and elicits a systemic and mucosal immune response to CTB. However, provision of R-CTB with mmCT selectively blunts the mucosal immune response to CTB. We propose that mmCT delivered by this live attenuated Vibrio cholerae vaccine platform may serve as a mucosal adjuvant for heterologous antigens, provided they are not too similar to mmCT.

**IMPORTANCE** Diarrheal disease is the most common infectious disease of children in the developing world. Our goal is to develop a diarrheal antigen presentation platform based on whole Vibrio cholerae cells that does not depend on protein purification. We have previously shown the feasibility of genetically fusing antigens to the V. cholerae biofilm matrix protein RbmA for presentation on the cell surface. A mucosal adjuvant could improve immunogenicity of such a vaccine at the mucosal surface. Here we engineer a live attenuated V. cholerae vaccine to constitutively synthesize mmCT, a nontoxic form of cholera toxin. When this vaccine is delivered sublingually, *in vivo*-synthesized mmCT acts as both an adjuvant and antigen. This could greatly increase the magnitude and duration of the immune response elicited by codelivered heterologous antigens.

## Abstract (precis)

Diarrheal disease is the most common infectious disease of children in the developing world. Our goal is to develop a diarrheal antigen presentation platform based on whole Vibrio cholerae cells that does not depend on protein purification. We have previously shown the feasibility of genetically fusing antigens to the V. cholerae biofilm matrix protein RbmA for presentation on the cell surface. A mucosal adjuvant could improve immunogenicity of such a vaccine at the mucosal surface. Here we engineer a live attenuated V. cholerae vaccine to constitutively synthesize mmCT, a nontoxic form of cholera toxin. When this vaccine is delivered sublingually, *in vivo*-synthesized mmCT acts as both an adjuvant and antigen. This could greatly increase the magnitude and duration of the immune response elicited by codelivered heterologous antigens.

## INTRODUCTION

Diarrhea is the leading infectious disease of young children in the developing world, and yet few vaccines targeting diarrheal disease exist (1). Our goal is to develop an antigen presentation platform for diarrheal disease that circumvents the need for protein purification. Vibrio cholerae is responsible for cholera, an epidemic diarrheal disease that targets children living in temperate climates and populations displaced by political unrest or environmental disaster, such as those in Yemen, Rwanda, and Haiti (2–4). Both live attenuated and inactivated whole-cell vaccines that protect against cholera have been developed and are licensed (5–7). Our laboratory has developed a technology that can transform these live attenuated or inactivated whole-cell cholera vaccines into vaccine platforms. This technology is based on genetic fusion of protein antigens to the biofilm matrix-associated protein RbmA (8).

Cholera toxin (CT), which produces the watery diarrhea characteristic of cholera (9), is quite similar to the heat-labile toxin (LT) of the diarrheal pathogen enterotoxigenic Escherichia coli (ETEC). While antilipopolysaccharide (anti-LPS) antibodies correlate most closely with protection against cholera (10), antibodies against the B subunit of cholera toxin (CTB) are thought to provide some protection against strains of ETEC that encode LT (11–13). We previously showed that plasmid-based expression of a genetic fusion of CTB to RbmA (R-CTB) resulted in abundant decoration of the cell surface with CTB (14). Sublingual delivery of such a vaccine elicited a mucosal immune response to CTB and provided passive protection against cholera challenge in an infant mouse model (8). However, expression of R-CTB from the native chromosomal location of RbmA resulted in decreased surface accumulation and poor immunogenicity. We hypothesized that a mucosal adjuvant might bolster immunogenicity (15–17). Our goal in this study was to incorporate a mucosal adjuvant into our antigen presentation platform. Both cholera toxin (CT) and a detoxified form of cholera toxin, mmCT (i.e., multiply mutated CT), are excellent mucosal adjuvants (18, 19). Both CT and mmCT have been shown to enhance vaccine efficacy by promoting differentiation and maturation of Th17 cells (20–22), which closely correlates with production of mucosal IgA (23). Here we engineer our vaccine platform to express mmCT after delivery to the sublingual mucosa. We show that *in vivo* delivery of mmCT by a live attenuated vaccine elicits a robust mucosal immune response to CTB and boosts the mucosal immune response to LPS. Coexpression of mmCT and an RbmA-CTB fusion does not improve the response to CTB but rather results in dose-dependent tolerance. We conclude that mmCT synthesized *in vivo* acts as a mucosal adjuvant for antigens presented by this live attenuated V. cholerae antigen presentation platform, provided they are sufficiently different from mmCT.

## RESULTS

### A live attenuated vaccine expressing a chromosomally encoded R-CTB variant elicits poor mucosal responses to both V. cholerae LPS and CTB.

**FIG 1:**
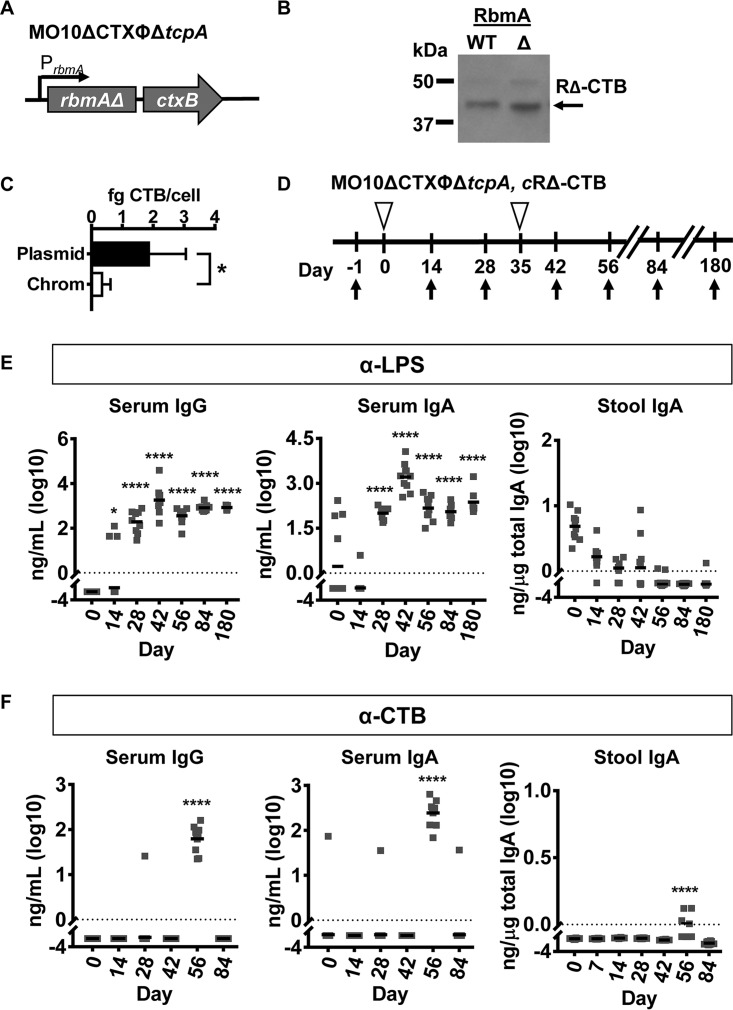
Chromosomally expressed RΔ-CTB is poorly immunogenic. (A) Genotype of an O139 serotype vaccine strain in which the entire CTX phage, including the recombination sites, as well as the *tcpA* gene, has been deleted. Two R→A mutations (R116A and R234A) were introduced into *rbmA*. CTB is genetically coupled to the 3′ end of *rbmA* and placed under the native *rbmA* promoter on the chromosome (cRΔ-CTB). (B) Western analysis of CTB fused to the C-terminal end of wild-type RbmA (WT) or an RbmA protein carrying the R116A and R234A point mutations (Δ) was detected in the V. cholerae cell pellet. (C) Quantification of cell-associated CTB in femtograms per cell when expressed from a plasmid or on the chromosome. (D) Vaccination scheme for the live attenuated vaccine. Open triangles indicate vaccination. Arrows indicate blood and stool sample collection. (E) Nanograms of LPS-specific antibodies per milliliter of serum or per microgram of total IgA in stool after immunization with a live attenuated vaccine strain expressing cRΔ-CTB. (F) Nanograms of CTB-specific antibodies per milliliter of serum or per microgram of total IgA in stool after immunization with a live attenuated vaccine strain expressing cRΔ-CTB. Error bars in panel C denote standard deviation. For panel C, * indicates *P* ≤ 0.05 using two-tailed, unpaired Student’s *t* test. For panels E and F, * indicates *P* ≤ 0.05 and **** indicates *P* ≤ 0.0001 using one-way ANOVA followed by Dunnett’s test for multiple comparisons. Horizontal bars mark the mean. Each vaccination group included 10 mice.

In our initial experiments, we used a plasmid encoding RbmA fused to CTB (8). To adapt our vaccine platform for safe delivery as a live attenuated vaccine, we moved the gene encoding R-CTB from a plasmid to the native RbmA site of a V. cholerae strain lacking both the entire CTX phage and *tcpA*, the major component of toxin-coregulated pilus (TCP) (Fig. 1A; see Tables S1 and S2 in the supplemental material) (24). Additionally, we introduced two R→A mutations in RbmA at positions 116 and 234 (RΔ-CTB). These mutations have previously been found to abrogate the rugose colony morphology (25). We hypothesized that these mutations might reduce cell-cell interactions mediated by RbmA and thus alter antigen delivery to immune cells. Indeed, we found that while RΔ-CTB remained associated with the cell pellet (Fig. 1B), biofilms formed under static conditions overnight by a strain carrying RΔ-CTB were more easily dispersed by agitation than a strain carrying wild-type R-CTB (see Fig. S1 in the supplemental material). This strain accumulated approximately 6-fold less RΔ-CTB on its surface than a strain that expresses CTB conjugated to wild-type RbmA encoded on a plasmid (Fig. 1C).

10.1128/mSphere.00245-18.5 TABLE S1  Strains and plasmids used in this study. Download TABLE S1, PDF file, 0.1 MB. Copyright © 2018 Liao et al. 2018 Liao et al. This content is distributed under the terms of the Creative Commons Attribution 4.0 International license.

10.1128/mSphere.00245-18.6 TABLE S2  Primers used for strain construction in this study. Download TABLE S2, PDF file, 0.1 MB. Copyright © 2018 Liao et al. 2018 Liao et al. This content is distributed under the terms of the Creative Commons Attribution 4.0 International license.

10.1128/mSphere.00245-18.1 FIG S1  Specific R116A and R234A mutations in RbmA abrogated tight cell-cell interaction mediated by RbmA in biofilms. Biofilms formed after static overnight incubation in LB broth by a strain carrying the R116A and R234A mutations in RbmA (RΔ-CTB) were dispersed by vortexing. The image is representative of experiments carried out in triplicate. Download FIG S1, PDF file, 0.2 MB. Copyright © 2018 Liao et al. 2018 Liao et al. This content is distributed under the terms of the Creative Commons Attribution 4.0 International license.

We delivered this strain sublingually to mice with one booster. Blood and stool were collected at the time points shown in Fig. 1D. While LPS-specific serum IgG and IgA were detected, low levels of LPS-specific IgA in the stool suggested that the mucosal immune response to LPS was poor (Fig. 1E). In addition, the systemic and mucosal immune responses to CTB were measurable only after boosting (Fig. 1F). We conclude that the vaccine strain carrying chromosomally encoded RΔ-CTB is poorly immunogenic compared with our previous observations of R-CTB expressed from a plasmid. We attribute this to decreased accumulation of RΔ-CTB on the cell surface. This led us to consider antigen delivery in tandem with a mucosal adjuvant.

### Immunization with an mmCT-producing vaccine results in a robust antibody response to CTB and augments the LPS-specific IgA response.

**FIG 2:**
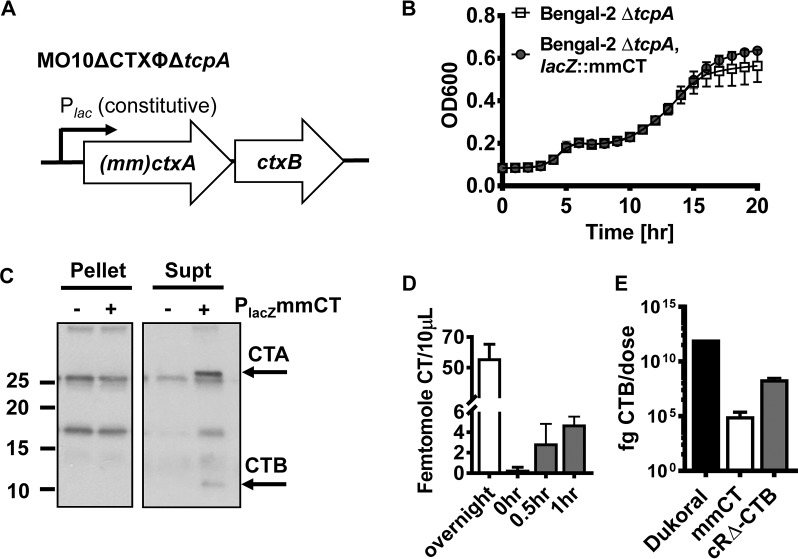
Characterization of mmCT as an *in vivo* antigen and adjuvant. (A) Genotype of an O139 serotype vaccine strain in which the entire CTX phage, including the recombination sites, as well as the *tcpA* gene has been deleted. The genes encoding mmCT (a cholera toxin variant carrying multiple mutations in the A subunit of cholera toxin) are integrated in frame into the *lacZ* gene on the V. cholerae chromosome. A ribosome-binding site is included at the 5′ end of the sequence encoding mmCT, allowing the *lacZ* promoter to drive transcription and translation of mmCT. (B) Constitutive production of mmCT in LB medium does not affect bacterial fitness as measured by growth over time. (C) Western blot analysis of cell pellets and supernatants (Supt) after overnight LB broth culture of the vaccine strain noted in panel A with or without mmCT. Rabbit sera raised against CT followed by secondary anti-rabbit antibody were used for detection. (D) Quantification of mmCT in the supernatant of an overnight culture in LB and after incubation of the vaccine preparation in PBS. Vaccines were kept on ice for less than 1 h before administration. (E) Quantification of the amount of CTB delivered as R-CTB and as part of mmCT, compared to the amount of purified CTB in Dukoral vaccine. The amount of CTB from supernatant mmCT was measured after 1 h of incubation at 22°C in PBS.

Cholera toxin (CT) is an excellent but impractical mucosal adjuvant due to its toxicity. Recently, a multiply mutated form of CT (mmCT) has been developed that retains adjuvanticity but has greatly reduced toxicity (19). We hypothesized that mmCT might serve as an excellent adjuvant for our vaccine. We introduced mmCT into the native *lacZ* site on the V. cholerae chromosome, where expression is driven by the *lacZ* promoter (P_*lacZ*_::mmCT) (Fig. 2A; see Table S3 in the supplemental material). Because the V. cholerae genome does not encode LacI, this promoter is constitutive in V. cholerae. mmCT would be suboptimal as an adjuvant if *in vivo* expression reduced cell fitness. However, we found that constitutive production of mmCT did not impair V. cholerae growth (Fig. 2B). We then established secretion of mmCT into the supernatant of bacteria cultured in Luria-Bertani (LB) broth by Western blotting using rabbit sera raised against cholera toxin (Fig. 2C). Using enzyme-linked immunosorbent assay (ELISA), we determined that a 10-µl volume of the cell-free supernatant from an overnight culture of the mmCT-producing strain contained approximately 50 fmol of mmCT. Furthermore, after incubation in phosphate-buffered saline (PBS) at room temperature for 30 min and 1 h, the length of time we estimate that the vaccine persists in the murine sublingual space, we measured approximately 3 fmol and 4.7 fmol of mmCT per 10-µl volume, respectively (Fig. 2D). Even after the 1-h incubation at room temperature in PBS, mmCT expression delivered approximately 4 orders of magnitude less CTB than is delivered by chromosomal expression of RΔ-CTB and 8 orders of magnitude less CTB than the Dukoral vaccine (Fig. 2E).

10.1128/mSphere.00245-18.7 TABLE S3  Oligonucleotides synthesized for strain construction. Download TABLE S3, PDF file, 0.1 MB. Copyright © 2018 Liao et al. 2018 Liao et al. This content is distributed under the terms of the Creative Commons Attribution 4.0 International license.

**FIG 3:**
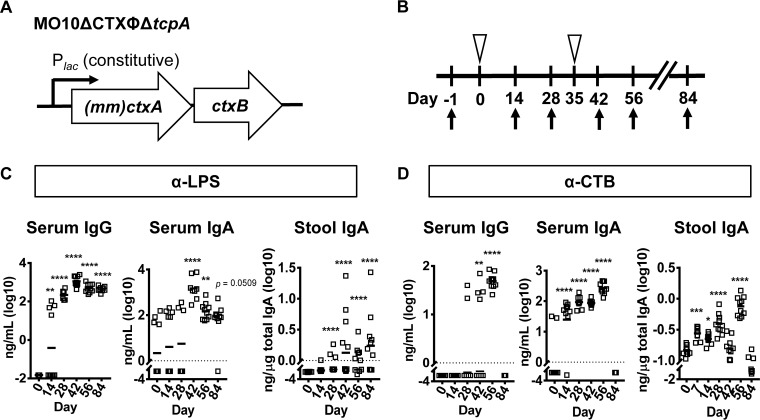
*In situ* production of mmCT alone after sublingual vaccine delivery elicits a robust and long-lived immune response to CTB. (A) Genotype of vaccine strain used. Expression of genes encoding mmCT is driven by the constitutive *lacZ* promoter on the chromosome of the vaccine strain. (B) Vaccination scheme for the live attenuated vaccines with constitutive expression of mmCT. Open triangles indicate vaccination. Arrows indicate blood and stool sample collection. (C) Nanograms of LPS-specific antibodies per milliliter of serum or per microgram of total IgA in stool after immunization with a live attenuated vaccine strain expressing mmCT. (D) Nanograms of CTB-specific antibodies per milliliter of serum or per microgram of total IgA in stool after immunization with a live attenuated vaccine strain expressing mmCT. *, *P* ≤ 0.05, **, *P* ≤ 0.01, ***, *P* ≤ 0.001, and ****, *P* ≤ 0.0001, using one-way ANOVA and Dunnett’s test. Horizontal bars mark the mean. Each vaccination group included 10 mice.

We administered the live attenuated vaccine expressing mmCT alone to mice via the sublingual route (Fig. 3A and B). Sublingual immunization with the live attenuated vaccine enhanced the mucosal immune response to LPS, which had not yet declined at day 84 after the primary immunization (Fig. 3C; see Fig. S2 in the supplemental material). Serum and mucosal immune responses to CTB were robust and also lasted for several weeks but declined by day 84 (Fig. 3D).

10.1128/mSphere.00245-18.2 FIG S2  The adjuvant mmCT enhances production of LPS-specific stool IgA. Shown are nanograms per milliliter of LPS-specific serum antibodies or nanograms per microgram of total IgA in stool measured 84 days after the first immunization with a live attenuated vaccine strain expressing chromosomal RΔ-CTB (cRΔ-CTB), mmCT from the constitutive *lacZ* promoter (mmCT), or both. **, *P* < 0.01 compared to the vaccine expressing chromosomal cRΔ-CTB without mmCT using one-way ordinary ANOVA followed by Dunnett’s multiple-comparison test. Horizontal bars mark the mean. Each vaccination group included 10 mice. Download FIG S2, PDF file, 0.1 MB. Copyright © 2018 Liao et al. 2018 Liao et al. This content is distributed under the terms of the Creative Commons Attribution 4.0 International license.

### Codelivery of mmCT and cRΔ-CTB attenuates the immune response to CTB.

**FIG 4:**
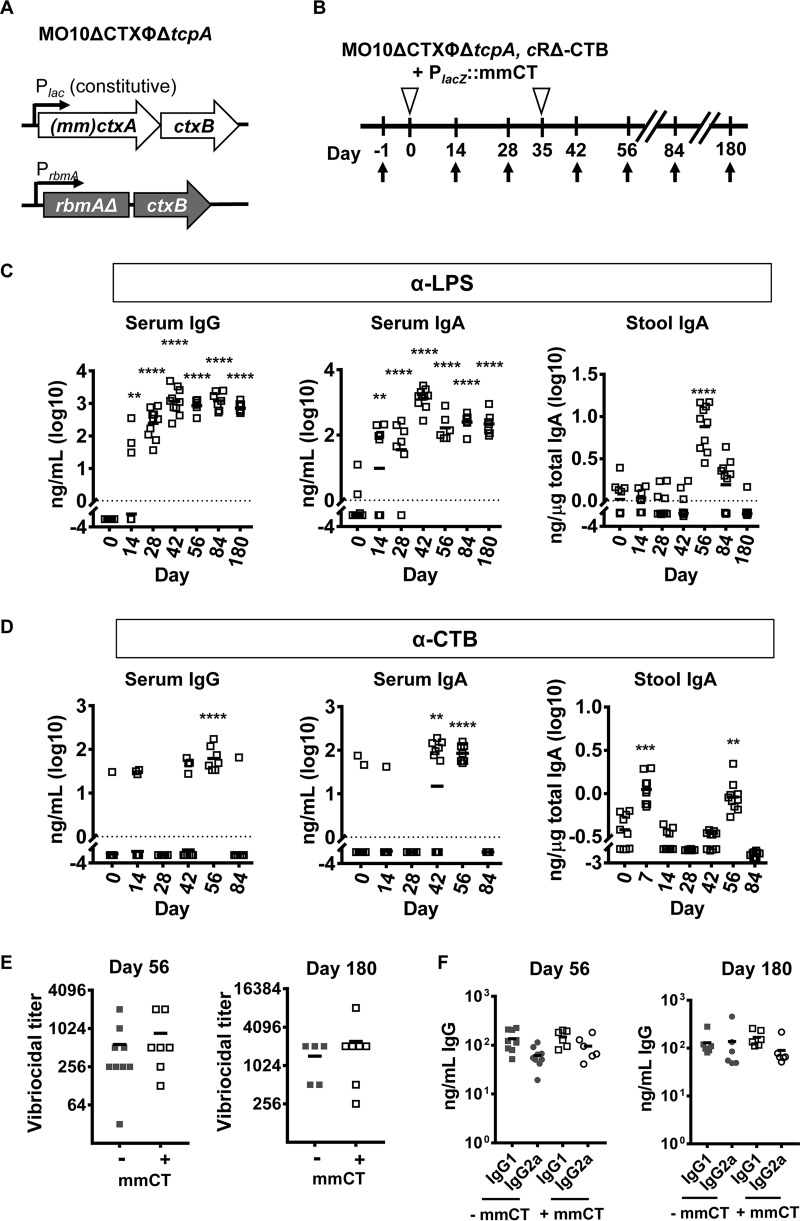
Coexpression of the adjuvant mmCT with chromosomal RΔ-CTB attenuates the immune response to CTB. (A) Genotype of an O139 serotype vaccine strain in which the entire CTX phage, including the recombination sites, as well as the *tcpA* gene has been deleted. Constitutive expression of genes encoding mmCT is driven by the native, chromosomal *lacZ* promoter. CTB is genetically coupled to the 3′ end of *rbmA* carrying R116A and R234A mutations and placed under the native *rbmA* promoter on the chromosome (cRΔ-CTB). (B) Vaccination scheme for the live attenuated vaccine with chromosomal RΔ-CTB and constitutive expression of mmCT. Open triangles indicate vaccination. Arrows indicate blood and stool sample collection. (C and D) Nanograms of LPS-specific antibodies per milliliter of serum or micrograms per total IgA in stool (C) and nanograms of CTB-specific antibodies per milliliter of serum or per microgram of total IgA in stool (D) after immunization with a live attenuated vaccine strain expressing cRΔ-CTB and mmCT. Data were log transformed prior to calculation of statistical significance. n.s., not significant (*P* ≥ 0.05), *, *P* ≤ 0.05, **, *P* ≤ 0.01, ***, *P* ≤ 0.001, and ****, *P* ≤ 0.0001, using one-way ANOVA and Dunnett’s multiple-comparison test. Horizontal bars mark the mean. Each vaccination group included 10 mice. (E and F) Comparison of (E) vibriocidal titers and (F) LPS-specific IgG1 and IgG2a for vaccines expressing cRΔ-CTB with or without mmCT at days 56 and 180. Differences were not statistically significant.

Given the adjuvant activity of mmCT, we questioned whether codelivery of chromosomally encoded cRΔ-CTB and mmCT might further improve the mucosal immune response to CTB. To test this, we administered a vaccine expressing cRΔ-CTB and mmCT on the chromosome to mice with one booster at day 35 (Fig. 4A and B). The combination of mmCT and cRΔ-CTB had a minimal effect on the immune response to LPS but attenuated the systemic and mucosal immune responses to CTB compared with the vaccine expressing only mmCT (Fig. 4C and D and Fig. S2). We hypothesized that this might reflect the development of tolerance due to the amino acid sequence shared between R-CTB and mmCT. To explore the impact of tolerance on the immune response to LPS, we measured vibriocidal antibodies, which correlate with protection against cholera. These were not significantly different at days 56 and 180 postvaccination for vaccines expressing RΔ-CTB with or without mmCT (Fig. 4E) and were comparable to the levels of vaccines previously demonstrated to confer protection against V. cholerae (8). Furthermore, LPS-specific IgG1 and IgG2a titers were similar between the two vaccines (Fig. 4F). To further confirm that this represented a tolerance response specifically to CTB, we assessed the impact of delivery of greater amounts of CTB through expression of R-CTB from a plasmid along with constitutive expression of chromosomally encoded mmCT (Fig. S3A). This vaccine was administered sublingually to mice according to the schedule shown in Fig. S3B, with boosters administered on days 14 and 28. Again, the immune response to LPS was similar to those of other vaccines expressing mmCT (Fig. S3C). However, the immune response to CTB was greatly attenuated (see Fig. S3D and S4 in the supplemental material). We conclude that mmCT is not an ideal adjuvant for heterologous proteins that are highly similar to cholera toxin, such as the heat-labile toxin of ETEC.

10.1128/mSphere.00245-18.3 FIG S3  Higher expression of R-CTB along with mmCT further dampens the immune response to CTB. (A) Genotype of an O139 serotype vaccine strain in which the gene encoding the A subunit of cholera toxin (*ctxA*) is deleted. Constitutive expression of genes encoding mmCT is driven by the native, chromosomal *lacZ* promoter. CTB genetically coupled to the 3′ end of wild-type *rbmA* is placed under a P_*tac*_ promoter on a multicopy plasmid (pR-CTB). (B) Vaccination scheme for the live attenuated vaccine with plasmid-encoded pR-CTB and constitutive expression of mmCT. Open triangles indicate vaccination. Arrows indicate blood and stool sample collection. (C and D) Nanograms of LPS-specific antibodies per milliliter of serum or per microgram of total IgA in stool (C) and nanograms of CTB-specific antibodies per milliliter of serum or per microgram of total stool IgA (D) after immunization with a live attenuated vaccine strain expressing plasmid-encoded pR-CTB and chromosomally encoded mmCT. Data were log transformed prior to calculation of statistical significance. n.s., not significant (*P* ≥ 0.05), ***, *P* ≤ 0.001, and ****, *P* ≤ 0.0001, using one-way ANOVA and Dunnett’s multiple-comparison test. Horizontal bars mark the mean. Each vaccination group included 10 mice. Download FIG S3, PDF file, 0.2 MB. Copyright © 2018 Liao et al. 2018 Liao et al. This content is distributed under the terms of the Creative Commons Attribution 4.0 International license.

10.1128/mSphere.00245-18.4 FIG S4  Inclusion of mmCT decreases CTB-specific serum and stool antibodies. Shown are nanograms per milliliter of CTB-specific serum antibodies or nanograms per microgram of total IgA in stool measured 56 days after the first immunization with live attenuated vaccines expressing chromosomal RΔ-CTB (cRΔ-CTB), mmCT from the constitutive *lacZ* promoter (mmCT), both cRΔ-CTB and mmCT, or wild-type R-CTB from a multicopy plasmid combined with mmCT (pR-CTB+mmCT). *, *P* < 0.05, and ****, *P* < 0.0001, compared to the vaccine expressing chromosomal cRΔ-CTB without mmCT using one-way ordinary ANOVA followed by Dunnett’s multiple-comparison test. Horizontal bars mark the mean. Each vaccination group included 10 mice. Download FIG S4, PDF file, 0.1 MB. Copyright © 2018 Liao et al. 2018 Liao et al. This content is distributed under the terms of the Creative Commons Attribution 4.0 International license.

## DISCUSSION

Adjuvanted vaccines that can be easily administered to prevent epidemic and endemic diarrheal disease will greatly improve quality of life in resource-poor settings. Our goal is to develop an antigen presentation platform that does not require purification of antigens or adjuvants. Here we describe the design of a live attenuated vaccine platform that is capable of synthesizing mmCT, its own mucosal adjuvant, *in vivo* and can be delivered sublingually. mmCT improves the magnitude and longevity of the mucosal immune response to V. cholerae LPS and elicits both systemic and mucosal responses to CTB. However, inclusion of CTB as an antigen fused to the biofilm matrix protein RbmA dampened the immune response to CTB in a dose-dependent fashion. We hypothesize that this is an antigen-specific tolerance response that results from the CTB peptide shared by R-CTB and mmCT. We conclude that while mmCT synthesized in the sublingual space can improve the immunogenicity of sublingually presented antigens, it is not an ideal adjuvant for antigens that are structurally similar to cholera toxin.

Here we have delivered mmCT through constitutive synthesis by a live attenuated vaccine administered via the sublingual route. The adjuvanticity of purified mmCT has previously been demonstrated (18, 19). A mechanism is suggested by studies of the double mutant LT (dmLT) adjuvant, an attenuated variant of the closely related heat-labile toxin of enterotoxigenic E. coli. Purified dmLT was shown to have adjuvant activity comparable to that of native cholera toxin when administered sublingually, likely by promoting proliferation of specific Th1 and Th17 cells (26–28). Detoxified cholera toxin derivatives have previously been incorporated into live attenuated V. cholerae strains and delivered via the nares (29). Similar to the sublingual route, the intranasal route of vaccine administration circumvents the requirement for passage through the acidic environment of the stomach, but also has unique disadvantages. Despite its documented safety in an oral formulation, intranasal administration of a dmLT-adjuvanted influenza vaccine was correlated with onset of Bell’s palsy in healthy adults (30–32). The safety of sublingually administered mmCT, which was developed as an alternative to dmLT, has not been investigated in humans. Therefore, future work will be necessary to establish the safety of mmCT in our current vaccine formulation.

The sublingual space is subject to frequent evacuations through deglutition. Nevertheless, for ease of administration, we have chosen to deliver our vaccine via this route. To increase residence time, thermoresponsive gels have developed for vaccine delivery to the sublingual space. Such a gel, when used for sublingual delivery of an inactivated polio vaccine combined with the adjuvant dmLT, improved production of mucosal IgA and neutralizing antibodies (33). Our data suggest that, in the absence of such a gel, our current live attenuated vaccine formulation induces robust systemic and mucosal immune responses. V. cholerae has a propensity to attach to diverse surfaces (34, 35). One advantage of our live attenuated vaccine vector may be its ability to attach to and interact with the sublingual surface, thus resisting evacuation during deglutition. Our findings warrant exploration of the interaction between our live attenuated vaccine and the sublingual mucosa and the impact of depot technologies on immunogenicity.

Here, we have produced a sublingually administered, adjuvanted live attenuated vaccine platform. This vaccine does not require additional preparation or protein purification, as the cholera toxin-based adjuvant is constitutively produced after administration. Furthermore, it induces a robust mucosal IgA response to Vibrio cholerae LPS and cholera toxin. This has implications for the development of future vaccines, especially those based on live attenuated V. cholerae vector strains. This innovation has the potential to simplify manufacturing and administration costs of vaccines effective against diarrheal pathogens.

## MATERIALS AND METHODS

### Bacterial strains and culture conditions.

Vibrio cholerae strains were cultured at 27°C in Luria-Bertani (LB) broth supplemented with 100 µg/ml streptomycin, with shaking at 200 rpm. Escherichia coli was grown in LB broth at 37°C with shaking. Where necessary, plasmids were maintained with 100 µg/ml of ampicillin in the culture medium. Protein production from plasmid-borne P_*tac*_ was induced with 0.5 mM IPTG (β-d-1-thiogalactopyranoside). Frozen stocks were maintained in 15% glycerol at −80°C. Strains used in this study are listed in Table S1.

### DNA manipulations and strain construction.

All oligonucleotides were synthesized by Integrated DNA Technologies, Inc., and are detailed in Tables S2 and S3. Restriction enzymes were purchased from New England Biolabs (NEB) and used according to the manufacturer’s instructions. Gibson assembly of DNA fragments was carried out with the NEBuilder Hi-Fi DNA assembly kit (NEB). PCRs were performed with GoTaq polymerase (Promega) for screening and Q5 high-fidelity polymerase (NEB) for cloning. Genomic DNA from wild-type strain MO10, a V. cholerae O139 serotype strain, was used as the PCR template unless otherwise noted.

### (i) Construction of vaccine strain harboring a deletion in *tcpA*.

A *sacB*-encoding suicide vector carrying a deletion construct for *tcpA* was introduced into MO10 ΔCTXφ by conjugation (36). Homologous recombination and positive deletion clone selection were carried out as described below.

### (ii) Construction of vaccine strain expressing plasmid-encoded R-CTB.

The previously described pFLAG-CTC derivative carrying the gene encoding the B subunit of cholera toxin (CTB) fused to the gene encoding wild-type RbmA (R-CTB) under an IPTG-inducible promoter was introduced into MO10 ΔCTXφ Δ*tcpA* by electroporation (14). Protein production in positive transformants was verified by Western blotting using an anti-CTB antibody as described below.

### (iii) Construction of chromosomal RΔ-CTB vaccine strain.

The *rbmA-ctxB* sequence was amplified from the pFLAG-CTC plasmid carrying R-CTB using the rbmA-RR primer series listed in Table S2. The two point mutations R116A and R234A were introduced by incorporation into the amplification primers. Gibson assembly was used to ligate the fragments into XhoI-linearized suicide plasmid pWM91. Plasmids from positive E. coli clones were isolated and confirmed by sequencing. Homologous recombination into the chromosomal *rbmA* gene of the MO10 ΔCTXφ Δ*tcpA* strain and positive clone selection were carried out as described below.

### (iv) Construction of P_*lacZ*_::mmCT in V. cholerae.

The mmCT sequence was obtained by Gibson assembly of PCR fragments amplified from genomic DNA, with the exception of the first 97 bases, which were amplified from an oligonucleotide synthesized to contain the proximal ribosome binding motif from the P_*trc*_ promoter and the first 97 nucleotides of the *ctxA* gene (Table S3). Mutations of mmCT (19) and stop codons distal to the ribosome binding site were introduced on primers (Table S2) and confirmed in the assembled product by sequencing. This was then ligated between two PCR fragments of the *lacZ* gene by Gibson assembly to obtain the final construct. This *lacZ*-mmCT construct was digested using SacI and ligated into the *sacB*-encoding suicide plasmid pWM91. Plasmids from positive E. coli clones were isolated, and the sequence of the inserted DNA was confirmed by sequencing with M13 primers. Homologous recombination into the MO10 ΔCTXφ Δ*tcpA* strain and positive clone selection were carried out as previously described (37, 38).

### Vaccine preparation. (i) Protein induction.

Frozen stocks of strains carrying the plasmid encoding R-CTB were inoculated into 3 ml of LB supplemented with streptomycin and ampicillin (LB-Sm/Amp) and cultured at 27°C overnight. This starter culture was collected by centrifugation, washed once with LB-Sm/Amp, and subcultured into 25 ml of fresh LB-Sm/Amp in a 250-ml flask. After incubation for 6 to 8 h at 27°C with shaking, IPTG was added to a final concentration of 0.5 mM, and the culture was incubated for an additional 2 h at 27°C with shaking.

### (ii) TCA precipitation of secreted proteins.

Proteins secreted into the supernatant were precipitated with trichloroacetic acid (TCA) and washed with acetone. Briefly, spent supernatant was passed through a 0.2-µm-pore filter to remove bacterial cells. TCA was added to the cell-free supernatant to a final concentration of 10%, and the supernatant was incubated overnight at 4°C with gentle mixing. Precipitated proteins were collected by centrifugation and washed three times with ice-cold acetone. Residual acetone was evaporated by brief incubation at 95°C. The protein pellet was resuspended in 4× Laemmli buffer containing β-mercaptoethanol and prepared for Western blotting as described below.

### (iii) Preparation of whole-cell vaccines.

After protein induction for strains carrying pR-CTB or after overnight culture for the strains encoding chromosomal RΔ-CTB vaccine constructs, the bacterial culture was centrifuged at 5,000 × *g* for 15 min at 4°C to collect cells. The supernatant was passed through a 0.2-µm-pore filter, and the resulting cell-free supernatant was used for TCA precipitation and Western blot analysis. The remaining bacterial pellet was washed three times with 12 ml of sterile PBS and finally resuspended in 1 ml of PBS. This constituted the live attenuated whole-cell vaccine. For each vaccine preparation, 10 µl was removed to quantify colony-forming units (CFU), and 20 µl was reserved for Western blot analysis. For each immunization, vaccines expressing mmCT were kept on ice for less than 1 h before administration, and all vaccines were used within 2 h of preparation. Each vaccine dose consisted of 10^8^ to 10^9^ cells in 10 µl.

### (iv) Western blot analysis.

Supernatants and cell pellet samples were separated by centrifugation. TCA precipitation of the supernatant was performed prior to detection of mmCT. These samples were combined with 4× Laemmli buffer containing β-mercaptoethanol, sonicated in an ice bath, boiled for 5 min, and briefly centrifuged to remove particulates. Proteins were resolved on a denaturing 4 to 20% gradient Tris-HCl gel and then transferred onto a polyvinylidene difluoride membrane by semidry transfer (BioRad). The membrane was blocked in Tris-buffered saline with 0.1% Tween (TBST) and 5% skim milk for 2 h at room temperature with gentle shaking. Fresh blocking solution containing primary antibody was added in a 1:1,000 dilution. Rabbit-derived serum raised against both the A and B subunits of cholera toxin (Sigma) was used to detect mmCT. After overnight incubation with primary antibodies, the membrane was washed 3 times with TBST. Membranes were then incubated for 2 h at room temperature with 1:5,000-diluted HRP-conjugated anti-rabbit secondary antibody (Cell Signaling) and developed using an ECL (enhanced chemiluminescence) Western blotting substrate (Pierce).

### (v) Quantification of R-CTB and RΔ-CTB by densitometry.

Known concentrations of purified CTB (List Laboratories) were resolved by SDS-PAGE alongside R-CTB or RΔ-CTB samples (plasmid and chromosomal) and used as standards for quantification. ImageJ was used to generate a standard curve fitted to the intensities of bands corresponding to the CTB standards. The concentrations of R-CTB and RΔ-CTB were calculated using the linear portion of the standard curve.

### (vi) Quantification of mmCT in supernatants by ELISA.

The amount of mmCT secreted into the supernatant by the live vaccine suspension was quantified by GM1 ELISA as previously described (39). The supernatant of a wild-type strain that does not express mmCT was similarly prepared and used as a negative control.

One hundred nanograms of bovine monosialoganglioside GM1 (Sigma) in sodium carbonate buffer was added to each well of a 96-well microtiter plate (Nunc, Maxisorp), incubated overnight at room temperature, and then washed in PBS-Tween (PBST). Serial dilutions of cell-free supernatants in PBS were applied to GM1-coated wells. Purified CTB was prepared in a 1-µg/ml concentration in PBS, and serial dilutions were also applied to GM1-coated wells to generate a standard curve. The plates were incubated overnight at room temperature, washed in PBST, and blocked in PBS-bovine serum albumin (BSA). Monoclonal anti-CTB IgG (Fisher no. PIMA183519) diluted to 1 µg/ml in PBST-BSA was added to the plates, and the mixture was incubated overnight. The plates were probed with HRP-conjugated goat anti-mouse antibodies (Bethyl Laboratories) and developed with Turbo 1-Step TMB (Thermo Fisher) as described below.

### Immunization and sample collection. (i) Animals.

Female 6- to 8-week-old BALB/c mice were used in all immunization experiments. Mice were purchased from Charles River Laboratories, Inc., and housed in a biosafety level 2 facility at Boston Children’s Hospital with food and water *ad libitum*. Mice were acclimatized for 5 days. All procedures had been previously approved by the Institutional Animal Care and Use Committee.

### (ii) Sublingual administration of live attenuated vaccine.

Mice were anesthetized by intraperitoneal injection with a cocktail of ketamine (100 mg/kg body weight) and xylazine (10 mg/kg) and then held upright, while 10 µl of the vaccine was delivered under the tongue by a micropipette directed toward the floor of the mouth. Mice were maintained in the upright position for 2 min before resting, ventral side down, for at least 30 min until regaining consciousness.

### (iii) Collection of blood and stool samples.

Blood and stool samples were collected 1 day before vaccination and at the designated time points throughout the study period. Fresh stool pellets were frozen at −80°C until use. Blood was collected from the tail vein using capillary tubes with clot activator (Sarstedt). Sera were obtained by clearing the clotted blood with centrifugation at 10,000 × *g* for 5 min at room temperature and stored at −20°C. Stool samples were prepared as previously described (40). Briefly, pellets were thawed on ice, transferred to 15-ml conical tubes containing 3 ml of chilled resuspension solution (0.1 mg/ml soybean trypsin inhibitor and a 3:1 mixture of PBS to 0.1 M EDTA), thoroughly homogenized, and centrifuged at 650 × *g* for 10 min at room temperature. The supernatant was collected and centrifuged once more at 15,300 × *g* for 10 min at 4°C. Phenylmethylsulfonyl fluoride (PMSF) was added to the supernatant to a final concentration of 2 mM. Stool samples were kept at −20°C or at −80°C for long-term storage.

### Enzyme-linked immunosorbent assays. (i) Quantification of CTB-specific antibodies by ELISA.

*Standard curve*. CTB-specific IgA was not available for use in a standard curve. Therefore, to assess linearity, standard curves were generated by capturing IgA and IgG in reference mouse serum with goat anti-mouse IgG or IgA. Microtiter plate wells were incubated overnight with 100 µg of goat anti-mouse IgG or IgA diluted in sodium carbonate buffer. The wells were washed in PBST and blocked with PBS-BSA. Reference mouse serum (Bethyl) was diluted to 1 µg/ml of total IgG or IgA and applied to the wells. The wells were washed after overnight incubation and then probed with HRP-conjugated goat anti-mouse antibodies and developed as described above for quantification of mmCT.

*Test samples*. Microtiter plates were coated with GM1 followed by purified CTB as described above. The plates were blocked in PBS-BSA and washed in PBST. Serially diluted sera or stool samples were applied to the wells and incubated overnight. Serum dilutions ranged from 1:50 to 1:6,400, and stool dilutions ranged from 1:2 to 1:128. The plates were probed and developed as described above. In addition, antigen-specific IgG1 and IgG2a antibodies were captured as described above and then detected with HRP-conjugated anti-IgG1 (Bethyl no. A90-105P) or anti-IgG2a (Bethyl no. A90-107P) antibodies.

*Instruments and data analysis*. Antigen-specific total IgA and IgG as well as total stool IgA were measured on a BioRad Benchmark Plus plate reader using a kinetic protocol and then analyzed using the built-in microplate Manager software (v5.2.1). LPS-specific serum IgG1 and IgG2a were measured on a Tecan Sunrise plate reader using a kinetic protocol and analyzed with the associated Magellan software (v7.2).

### (ii) Quantification of total stool IgA by ELISA.

Total fecal IgA was used to normalize antigen-specific IgA in the stool. Each well of a microtiter plate was coated with 100 ng of goat anti-mouse IgA antibody in sodium bicarbonate buffer and incubated overnight. Plates were washed in PBST and blocked in PBS-BSA. Stool samples were serially diluted from 1:200 to 1:25,600 in PBST-BSA and added to the plates. The plates were incubated overnight and then probed and developed as described above. Standard curves were generated as described for CTB/LTB-specific antibodies.

### (iii) LPS extraction and measurement of O-antigen-specific antibodies.

LPS was extracted from 50 ml of a V. cholerae MO10 (serotype O139) overnight culture using a commercial kit (Bulldog Bio). Serum and stool antibodies recognizing the O1 or O139 serotypes were quantified as previously described (41). A 1:1,000 dilution of LPS in sodium carbonate buffer was applied to microtiter plates and incubated overnight. The plates were washed in PBST and blocked for 40 min at 37°C in PBS-BSA. Serum and stool samples were applied to the plates in dilutions similar to those used to measure CTB-specific antibodies. The plates were incubated for 90 min at 37°C and then washed in PBST. Plates were incubated for 90 min at 37°C after addition of 100 ng of HRP-conjugated goat anti-mouse antibodies per well. Plates were developed using the same protocol described for quantification of CTB-specific antibodies.

### Vibriocidal antibody titers.

Serum vibriocidal antibody titers were determined as previously described with the following modification (42). Mouse sera collected prior to and on the indicated days after immunization were incubated at 56°C for 1 h to inactivate endogenous complement, serially diluted 2-fold in PBS in 0.5-µl tubes, and then kept on ice until use. Wild-type MO10 was grown to mid-logarithmic phase in brain heart infusion (BHI) broth containing 100 µg/ml streptomycin and diluted in PBS containing 20% guinea pig complement to 4 × 10^6^ CFU/ml. An equal volume of this suspension was added to the serum dilutions to obtain a final concentration of 10% complement and 2 × 10^6^ CFU/ml V. cholerae, and the mixture was incubated for 1 h at 37°C. Five microliters of this mixture was inoculated into wells of a 384-well microtiter plate containing 35 µl of BHI with streptomycin and incubated at 37°C for 4 h. At this point, growth was assessed by measuring the optical density at 600 nm (OD_600_). The reciprocal of the highest serum dilution at which the OD_600_ remained below the level of detection was noted as the bactericidal titer.

### Ethics statement.

Animal experiments were performed in accordance with standards outlined in the National Research Council’s *Guide for the Care and Use of Laboratory Animals* and Boston Children’s Hospital’s Public Health Service-approved Animal Welfare Assurance. The protocol was approved by the Boston Children’s Hospital Institutional Animal Care and Use Committee (IACUC) appointed to review proposals for research involving vertebrate animals.

### Statistical analysis.

Statistical analyses were performed in GraphPad Prism 7. One-way ordinary analysis of variance (ANOVA) with Dunnett’s test was used for multiple comparisons. A two-tailed, unpaired Student’s *t* test was used for pairwise comparisons. All vaccine groups consisted of 10 mice. Error bars indicate standard deviations unless otherwise noted. Western blots and photographic images are representative of experimental triplicates.
