# Supplementary material for: Sublingual Adjuvant Delivery by a Live Attenuated Vibrio cholerae-Based Antigen Presentation Platform
Source: mSphere. 2018 Jun 6;3(3):e00245-18. doi: 10.1128/mSphere.00245-18 (PMC5990885; doi:10.1128/mSphere.00245-18)
Supplement: TABLE S1 [file sph003182561st1.pdf]

Table S1. Strains and plasmids used in this study.

Strains / Plasmid	Description	Reference
V. cholerae		
PW839	MO10Δ*ctxA*; Sm^r	(1)
PW1947	MO10Δ*ctxA,* P_{lacZ}mmCT; Sm^r	This study
PW139 (Bengal-2)	MO10Δ*attRS1*; Sm^r	(2)
PW1159	MO10Δ*attRS1*Δ*tcpA*; Sm^r	This study
PW1848	MO10Δ*attRS1*Δ*tcpA*, P_{lacZ}mmCT; Sm^r	This study
PW1843	MO10Δ*attRS1*Δ*tcpA*, P_{rbmA}RΔ-CTB; Sm^r	This study
PW1850	MO10Δ*attRS1*Δ*tcpA*, P_{rbmA}RΔ-CTB, P_{lacZ}mmCT; Sm^r	This study
E. coli		
SM10λpir	*thi thr leu tonA lacY supE recA*::RP4-2-Tc::Mu (λpirR6K); Km^r	(3)
**Plasmids**		
pWM91	oriR6K mobRP4 *lacI* pTac *tnp* mini-Tn*10*; Km^r Ap^r	(4)
pWM91-*lacZ*::mmCT	pWM91 carrying in-frame mmCT insertion into *lacZ* for homologous recombination; Ap^r	This study
pWM91-R-CTB	pWM91 carrying in-frame R-CTB insertion into native *rbmA* locus for homologous recombination; Ap^r	This study
pFLAG-CTC-*rbmA-ctxB*	IPTG-inducible expression of RbmA-CTB fusion protein; Ap^r	(5)
pHT3	pCVD442 carrying unmarked, in-frame delection of *tcpA*; Ap^r	(6)

References

1. Berkey CD, Blow N, Watnick PI. 2009. Genetic analysis of *Drosophila melanogaster* susceptibility to intestinal *Vibrio cholerae* infection. Cellular Microbiology 11:461–474.
2. Waldor MK, Mekalanos JJ. 1994. Emergence of a new cholera pandemic: molecular analysis of virulence determinants in *Vibrio cholerae* O139 and development of a live vaccine prototype. The Journal of Infectious Diseases 170:278-283.
3. Miller VL, Mekalanos JJ. 1988. A Novel Suicide Vector and Its Use in Construction of Insertion Mutations: Osmoregulation of Outer Membrane Proteins and Virulence Determinants in *Vibrio cholerae* Requires *toxR*. Journal of Bacteriology 170:2575-2583.
4. Metcalf WW, Jiang W, Daniels LL, Kim S-K, Haldimann A, Wanner BL. 1996. Conditionally replicative and conjugative plasmids carrying *lacZa* for cloning, mutagenesis, and allele replacement in bacteria. Plasmid 35:1-13.
5. Absalon C, Ymele-Leki P, Watnick PI. 2012. The bacterial biofilm matrix as a platform for protein delivery. mBio 3:e00127-12-e00127-12.
6. Thelin KH, Taylor RK. 1996. Toxin-Coregulated Pilus, but Not Mannose-Sensitive Hemagglutinin, Is Required for Colonization by *Vibrio cholerae* O1 El Tor Biotype and O139 Strains. Infection and Immunity 64:2853–2856.
