# Supplementary material for: Sublingual Adjuvant Delivery by a Live Attenuated Vibrio cholerae-Based Antigen Presentation Platform
Source: mSphere. 2018 Jun 6;3(3):e00245-18. doi: 10.1128/mSphere.00245-18 (PMC5990885; doi:10.1128/mSphere.00245-18)
Supplement: TABLE S2 [file sph003182561st2.pdf]

Table S2. Primers used for strain construction in this study.

Primer	Sequence[a]
**P_{lacZ}::mmCT**	
mmCT_1F	GATCATTTGG**TAATAG**GTATCGATTAAAT*AAGGAG*G
mmCT_1R	TGCTTTATTTCGTCGGGCGGGCGACTATC
mmCT_2F	CCGCCCGACGAAATAAAGCAGTCAGGTGGTCTTATGC
mmCT_2R	ATAACCATCTGCTGCTGGAGCAATATCTAAGTTACTG
mmCT_3F	ATTGCTCCAGCAGCAGATGGTTATGGATTGGCAGGTTTC
mmCT_3R	ATCACCCGTGATTGTTCCGCTACTATCCCCACAACCCGGCGGTGCATGATG
mmCT_4F	GATAGTAGCGGAACAATCACGGGTGATACTTGCGATGAAAAAACCCAAAGTC
mmCT_4R	GATTGGTATTCGTCAGCGAATTTTACACCTAGACTTTG
mmCT_5F	GTAAAATTCGCTGACGAATACCAATCTAAAGTTAAAAGAC
mmCT_5R	GTATTGCACAGGTTAATTTGCCATACTAATTGCG
lacZ_1F	GCGCGCGC*GAGCTC*AAGCCTTACATACAGGCCAGCG
lacZ_1R	CGATAC**CTATTA**CCAAATGATCACACAAGGGTG
lacZ_2F	GCAAATTAACCTGTGCAATACGAAGGGGGC
lacZ_2R	GCGCGCGC*GAGCTC*GCTGGACTTTTTTGACTTCATGTAATG
**rbmA(R116A, R234A)-ctxB**	
rbmA-RR_1F	ATTGGGTACCGGGCCCCCCCGCCTTAGCGCCAGTTGTAAAAAC
rbmA-RR_1R	TGCTGTAACGTTCAACATACGACCATCAGTAAGAG
rbmA-RR_2F	ATGGTCGTATGTTGAACGTTACAGCAGGTTTC
rbmA-RR_2R	GGATAGCTTTTATCAAAATTAATACCTGCCACTTTTG
rbmA-RR_3F	GCAGGTATTAATTTTGATAAAAGCTATCCAGCGGGCG
rbmA-RR_3R	ATCGATACCGTCGACCTCGAAAAGTCAATATAAAGCCATTATTAGAAC

[a] Start and stop codons are shown in boldface, sequence for the ribosome binding site is shown in italics. Relevant restriction site sequences are underlined and italicized. Mutations introduced in the sequence encoding mmCT are underlined and specific point mutations are colored in red.
