# Supplementary figures and images for: Sublingual Adjuvant Delivery by a Live Attenuated Vibrio cholerae-Based Antigen Presentation Platform
Source: mSphere. 2018 Jun 6;3(3):e00245-18. doi: 10.1128/mSphere.00245-18 (PMC5990885; doi:10.1128/mSphere.00245-18)

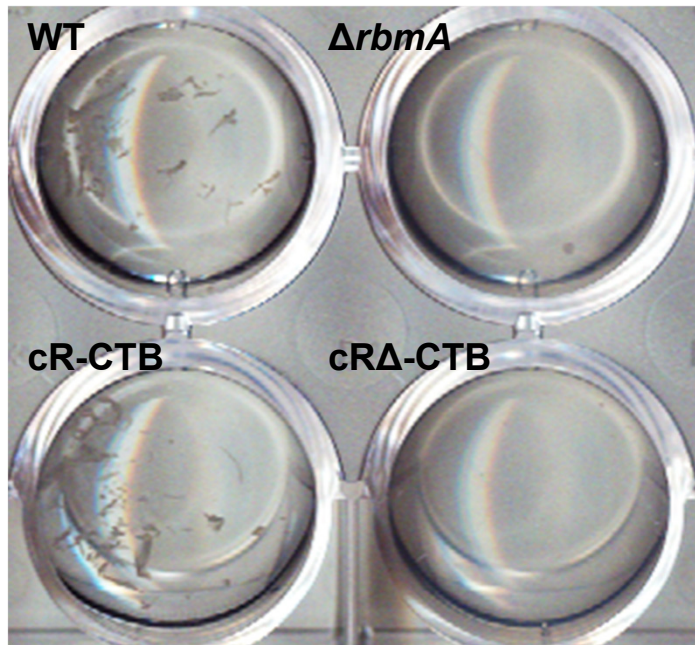

**Figure S1**

Supplement: FIG S1 [file sph003182561sf1.pdf]

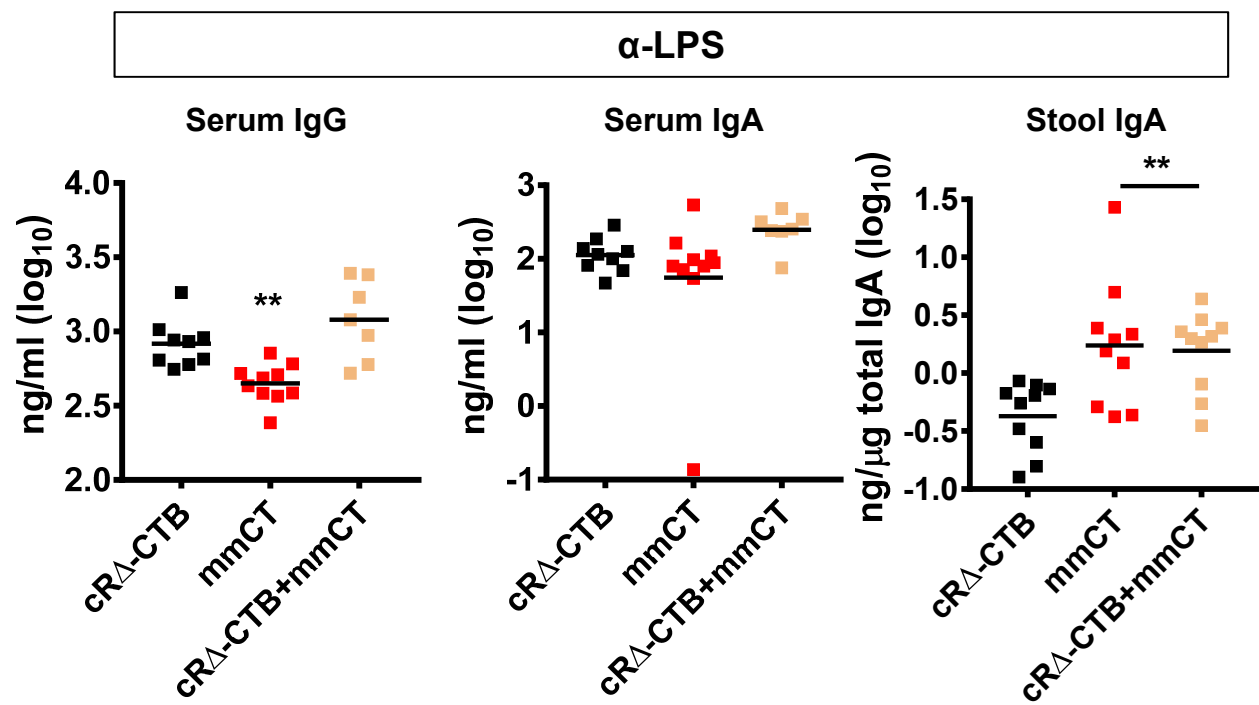

Figure S2

Supplement: FIG S2 [file sph003182561sf2.pdf]

# A MO10ΔctxA

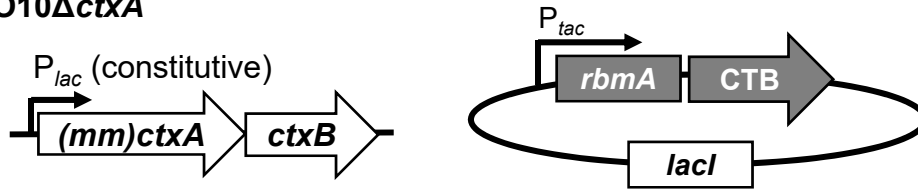

# B

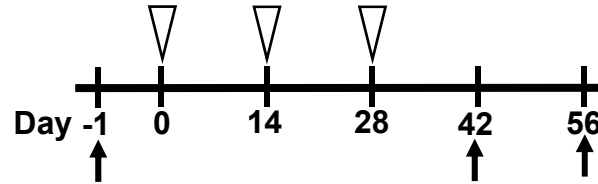

# C

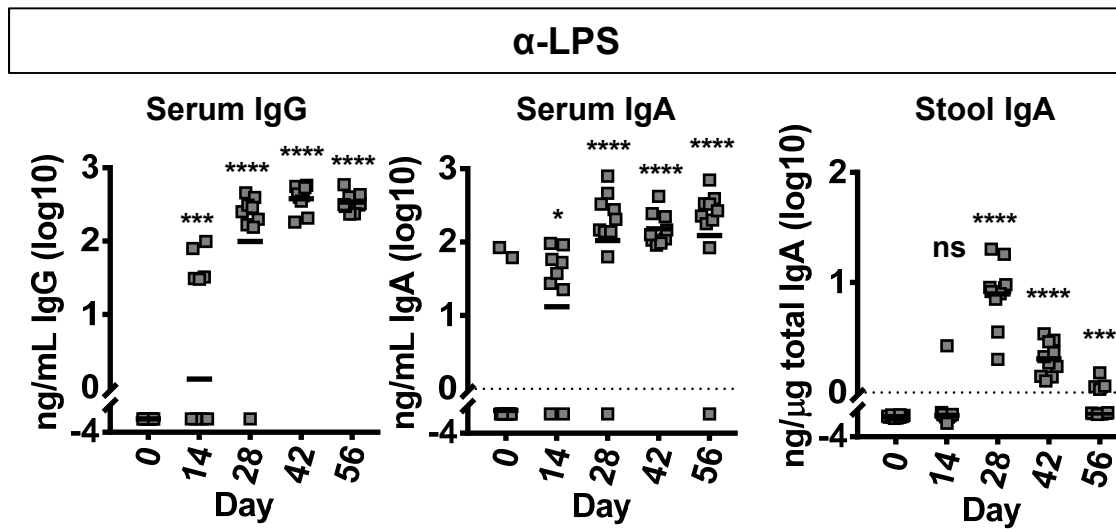

# D

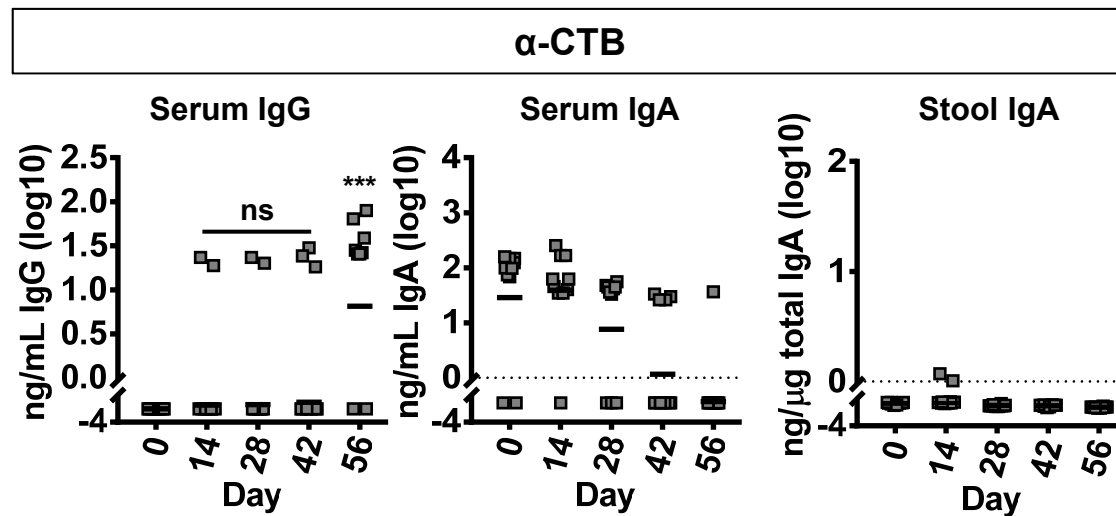

Supplement: FIG S3 [file sph003182561sf3.pdf]

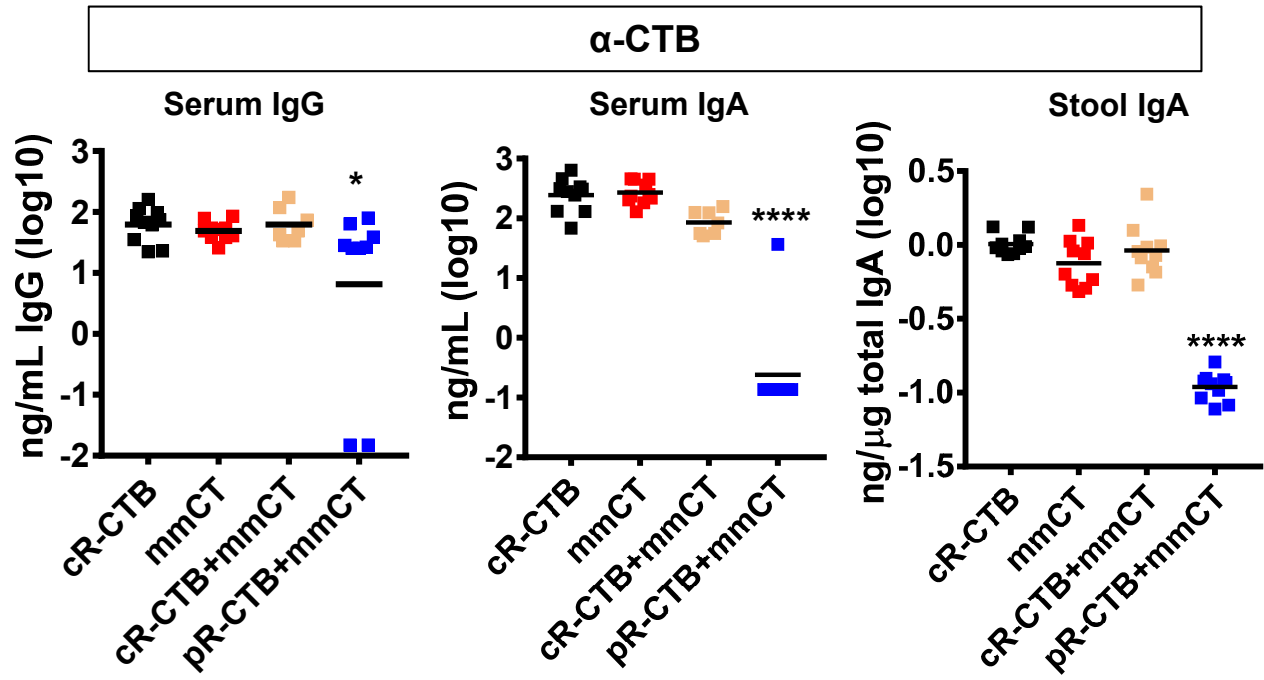

Figure S4

Supplement: FIG S4 [file sph003182561sf4.pdf]
