# Supplementary material for: Sublingual Adjuvant Delivery by a Live Attenuated Vibrio cholerae-Based Antigen Presentation Platform
Source: mSphere. 2018 Jun 6;3(3):e00245-18. doi: 10.1128/mSphere.00245-18 (PMC5990885; doi:10.1128/mSphere.00245-18)
Supplement: TABLE S3 [file sph003182561st3.pdf]

Table S3. Oligonucleotides synthesized for strain construction.

Construct[a]
**P*lacZ*::mmCT**
GTATCGATTAAAT*AAGGAG*GAATAAACCATGGTAAAGATAATCTTCGTGTTCTTCATCTTCCTGAGCAGCTTTTCGTACGCTAACGATGATAAGCTCTATCGCGCAGATAGTCGCCCGCCCGACG

[a] Ribosome binding site AAGGAG shown in italics
